# Risk Stratification of Chronic Myeloid Leukemia According to Different Prognostic Scores

**DOI:** 10.7759/cureus.7342

**Published:** 2020-03-20

**Authors:** Javeria Aijaz, Nada Junaid, Muhammad Asif Naveed, Rafeeda Maab

**Affiliations:** 1 Pathology, King Edward Medical University, Lahore, PAK; 2 Hematology, Punjab Institute of Thalassemia Prevention, Lahore, PAK; 3 Pathology, Jinnah Hospital/Allama Iqbal Medical College (AIMC), Lahore, PAK

**Keywords:** prognosis, cml, scores, sokal, hasford, eutos

## Abstract

Introduction

Several prognostic indices are in use to stratify chronic myeloid leukemia (CML) patients: Sokal, Hasford, and the European Treatment and Outcome Study (EUTOS) being the most commonly reported ones. The application of different scores may cause variability in the determination of disease prognosis. This study was conducted to stratify patients of CML in accordance with Sokal, Hasford, and EUTOS scoring systems and to determine the concordance rate of risk categories, calculated by using all three scoring systems.

Methods

This study was conducted at King Edward Medical University from January 2013 to May 2019. A total of 114 patients were diagnosed with CML in the chronic phase during the study period and included in the analysis. Variables of interest were computed using Microsoft Excel. These variables include age, spleen size, platelet count, the percentage of myeloblasts in the peripheral blood, as well as the percentage of basophils and eosinophils in the peripheral blood. Using these baseline variables, the prognostic category of each patient was calculated using Sokal, Hasford, and EUTOS scores.

Results

The male to female ratio of patients included in the study was 1.43. The mean age was 39.3±1.58 years, with an age range of 13 to 95 years. A total of only 4 out of 73 patients were categorized as a low-risk category, whereas 23 out of 80 patients were categorized into a high-risk category by all three scoring systems. The assignment of prognostic categories was variable, depending on which prognostic score was applied. The concordance rate of Sokal vs Hasford was 53%, Sokal vs EUTOS 64%, and Hasford vs EUTOS 98%.

Conclusion

There is considerable inter-variability between the various prognostic indicators. In general, the Hasford and EUTOS scores assign some patients to a lower risk category when compared to Sokal score.

## Introduction

The treatment outcome of Philadelphia-positive chronic myeloid leukemia (CML) patients has improved a lot after the start of the use of tyrosine kinase inhibitors (TKI). Determination of disease prognosis, however, is largely based on traditional prognostic models. These traditional models were developed when modern TKIs were not present and physicians use to treat patients with hydroxyurea, etc. There is no advancement in the development of a comprehensive prognostic score, which may also cater TKI response. Traditional prognostic models are currently in use, not only in studies carried out in patients on first-line TKIs but also in patients on second-generation TKIs [1].

Prognostic indices are calculated using various combinations of baseline variables including the age of the patient, the size of the spleen, platelet count, and percentages of myeloblasts, basophils, and eosinophils in the peripheral blood. The two commonly reported and used scoring systems include the Sokal score, introduced in 1984 to stratify hydroxyurea-treated patients into risk groups and the Hasford score, proposed by Hasford and co-workers in 1998 for interferon-treated patients [2-3]. These scores stratify patients into three risk groups: low-risk (LR), intermediate-risk (IR), and high-risk (HR) [2-3].

The European Treatment and Outcome Study (EUTOS) scoring system, devised in 2011, is the only prognostic system developed during the imatinib era [4]. The score identifies 2 risk groups: low-risk (LR) and high-risk (HR). Utilizing the percentage of basophils and spleen as the baseline variables for its computation, the score nevertheless remains to be validated in large studies. Current prognostic indices are used interchangeably in various settings, though the EUTOS score has not yet entered widespread clinical practice. Large-scale studies comparing the predictive utility of these indices have not been conducted, though some small-scale studies show discordant results. Moreover, no published study has compared the risk stratification concordance rate of all three scores against one another.

We conducted this study to categorize patients using Sokal, Hasford, and EUTOS scoring systems and we shall also determine the concordance rate of risk categories used by these scoring systems. 

## Materials and methods

This study was conducted at King Edward Medical University from January 2013 to May 2019. A total of 114 patients were diagnosed with CML in the chronic phase during the study period and included in the analysis. Variables of interest were computed using Microsoft Excel. These variables include age, spleen size, platelet count, the percentage of myeloblasts in the peripheral blood, as well as the percentage of basophils and eosinophils in the peripheral blood. Using these baseline variables, the prognostic category of each patient was calculated using Sokal, Hasford, and EUTOS scores.

The Sokal worked on 813 patients having CML in chronic phase, and after multivariate analysis, he derived a formula to calculate Sokal score. He worked between 1962 and 1981 for this purpose. Majority of the patients were treated by busulphan at that time. The formula of the score is as follows: exp (0.0116 x (age [years] − 43.4)) + (0.0345 x (spleen size [cm] − 7.51) + (0.188 x ((platelets [109/L]/700)^2 − 0.563)) + (0.0887 x (blasts [%] − 2.10)). Sokal *et al*. proposed three risk groups: low-risk (Sokal score < 0.8, 39% of patients), intermediate-risk (Sokal score 0.8-1.2, 38% of patients) and high-risk (> 1.2, 23% of patients) [2].

Later, in 1998, Hasford published a paper describing Hasford score or Euro score. This score was the result of multivariate analysis on 981 patients of CML. Majority of these patients were treated by interferon alpha alone, or in combination with another therapy. The study was conducted on patients enrolled between 1983 and 1994. A cohort of 322 patients was selected to validate the results. The score is calculated using the following formula: (0.6666 x age [0 when age < 50 years; 1 otherwise]) + (0.0420 x spleen size [cm]) + (0.0584 x blasts [%]) + (0.0413 x eosinophils [%]) + (0.2039 x basophils [0 when basophils < 3%; 1 otherwise]) + (1.0956 x platelet count [0 when platelets < 1500 x 109/L; 1 otherwise]) x 1000). Three risk groups were identified: low-risk (score ≤ 780, 40.6% of patients), intermediate-risk (score 781 - 1480, 44.7% of patients) and high-risk (score ≥ 1481, 14.6% of patients) [3].

The European Treatment and Outcome Study (EUTOS) risk score for CML was a product of larger sample size, i.e. 2060 patients. These patients were treated with imatinib between 2002 and 2006. The score is meant to apply at the time of diagnosis and before the start of therapy. The EUTOS score is calculated as (7 x basophil [%]) + (4 x spleen [cm]). Two risk groups were identified: low-risk (score <87, 79% of patients) and high-risk (score ≥87, 21% of patients). The EUTOS manuscript also provides the following formula for predicting the probability of no complete cytogenetic remission (CCgR) at 18 months: exp(−2.1007 + 0.0700 × basophils + 0.0402 × spleen size)/(1 + exp[−2.1007 + 0.0700 + basophils + 0.0402 × spleen size]) [4].

## Results

A total of 114 patients were enrolled in the study having a male to female ratio of 1.43. The mean age was 39.3±1.58 years, with an age range of 13 to 95 years. The mean spleen size below the costal margin was 14.7±6.5 cm. The percentages of basophils, blasts, platelets, and eosinophils were 2.3 ± 1.9, 6.9 ± 8.1, 403.5 ± 294.4, and 3.6 ± 3.0, respectively.

By Sokal score, 4 out of 114 patients were categorized as LR, 29 out of 114 patients as IR and 81 out of 114 patients as HR. By Hasford score, however, 16 out of 114 patients were classified as LR, 60 out of 114 patients as IR, and 38 out of 114 patients as HR. By EUTOS score, 74 out of 114 patients were classified as LR and 40 out of 114 patients as HR. The percentages of patients falling under the various risk categories, as determined by applying all three scores, are given below (Table 1).

**Table 1:** Percentage of the total sample size (n = 114) categorized as LR, IR and HR by applying Sokal, Hasford, and EUTOS scores, respectively

	Sokal Score	Hasford Score	EUTOS Score
Low Risk	4%	14%	65%
Intermediate Risk	25%	53%	-
High Risk	71%	33%	35%

In the LR category (defined as LR by one or more scores), only 4/74 patients were uniformly classified by all three scores. The IR category is not defined by the EUTOS score. Therefore, equating the IR of the Sokal and Hasford scores with the LR of the EUTOS score, only 33/74 patients were classified as LR and/or IR by all three scores. In the IR (defined as IR by one or more Scores) category, only 18/65 patients were classified as IR by both Hasford and Sokal Scores. In the HR category, only 23/80 patients were uniformly classified as HR. The overall percentage concordance of risk categories, when calculated by all three scores on the same patient population, is given in the table below (Table 2). For comparison with the EUTOS score, the IR of Sokal and Hasford scores are included with the LR.

**Table 2:** Percentage concordance of Sokal, Hasford and EUTOS scores

	Sokal vs Hasford	Sokal vs EUTOS	Hasford vs EUTOS vs Eutos Score
Concordance	53%	64%	98%

## Discussion

The mean age of patients in the present study, at 39.3±1.58 years, is much lower than that observed in many Western countries but is similar to other studies conducted in South Asia [5-6]. Differences in age at diagnosis have been reported within and between regions. In general, underdeveloped and developing countries have a lower age at presentation as compared to developed countries. Asia and Africa, continents comprising mostly underdeveloped and developing countries, have a much lower mean age at presentation compared to Sweden, a developed country with a mean age of 60 years at presentation [7-8] Additional epidemiological studies need to be conducted to assess for possible environmental factors accounting for earlier age at onset in developing countries like Pakistan. The difference could, nevertheless, be due to demographic characteristics of developing countries with lower mean age, lower life expectancy, and a greater proportion of the young population.

The present study shows that Hasford and EUTOS scores correlate well when used to stratify CML patients according to risk categories. Only 2% of risk categories assigned by these scores were discrepant. Comparison of risk categorization by using Sokal with the other two scores (Hasford and EUTOS), however, showed marked discrepancies. Approximately 47 % of the Sokal and Hasford scores were discrepant, whereas 36% of Hasford and EUTOS scores were also discordant. Also, the Sokal score generally assigns patients to a higher risk category as compared to Hasford and EUTOS scores. Most of the patients, when categorized by the Hasford score, fall under the low- and intermediate-risk categories. The same is true of the EUTOS score, which, however, does not have an intermediate risk category.

A study done in India by Sinha SK *et al*. has also reported the percentages of patients falling under respective risk categories, using the Sokal and Hasford scores [8]. The percentage of patients falling under the respective risk categories using the Sokal score in this study, however (LR: 18%, IR: 48%, and HR: 34%), is markedly different from the present one. Similarly, the application of Hasford score in this study also has produced risk categorization that is different from the present study (LR: 32%, IR: 50%, and HR: 18%). Nonetheless, this study too shows that more patients are placed in the high-risk category when using the Sokal score (34%), than when using the Hasford score (18%) [9]. The study by Oyekunle AA et al in Nigeria also reported a higher percentage of patients falling under the high-risk category when categorized by the Sokal Score than when categorized by Hasford Score [10]. The difference is, however, smaller than that found in the present study (Sokal: LR-40.3%, IR-33.6% and HR-26.1%, Hasford: LR-53.7%, IR-36.6%, and HR-9.7%). Another study from Pakistan by Syed NN *et al.* assigned lesser patients to the high-risk Sokal score category (46%) as compared to the present study [11]. Yet another study from Pakistan, however, by Usman M *et al*. reported only 3.7% of patients in the LR category versus 27.4% in the IR and 67.7 % in the HR categories [6]. The percentages reported in this later study are more in concordance with the percentages of risk categories calculated using the Sokal score in the present one.

The present study thus demonstrates that there is marked inter-variability in risk categorization when the various prognostic indicators for risk stratification of CML are applied to the same patient population. This is especially so when the most commonly used - Sokal score - is compared to either Hasford or EUTOS scores. In general, Hasford and EUTOS scores assign patients to a lower risk category as compared to the Sokal score. Under the assumption that varied demographic characteristics in different CML patient populations mean different inherent disease characteristics, which may, in turn, also have a bearing on the variables used for risk categorization; comparisons of the results of the present study have been done with other studies conducted in the region (South Asia) or with other countries having similar demographic profiles of CML patients (Nigeria). These comparisons demonstrate the marked variability of proportions of risk categories of CML patients when calculated using the traditional prognostic scores. One common finding in all studies, however, is that Sokal score places patients under higher risk categories than Hasford score.

## Conclusions

Determination of prognosis in CML patients has a bearing on the management of the disease. Disease prognosis, therefore, needs to be reliably determined by a valid method. Application of different indices to determine disease prognosis may not only impact the reliability of risk stratification but may also make inter-institutional comparisons difficult. Taking into consideration the marked discordance in risk stratification of the present study when using different scores, the variability in the reported percentages of patients falling under the respective risk categories in different studies using the same prognostic score; and the fact that systematic studies have been done to show that in the current era of PTK1 therapy, the most important indicator is the response to treatment at the hematologic, cytogenetic, and molecular level, we need to devise the new scoring system incorporating these more predictive criteria.
